# Multiple human papillomavirus infection and high-grade cervical squamous intraepithelial lesions among women with human immunodeficiency virus: a systematic review and a meta-analysis

**DOI:** 10.3389/fmed.2024.1403548

**Published:** 2024-07-15

**Authors:** Chiara Cassani, Mattia Dominoni, Marianna Francesca Pasquali, Barbara Gardella, Arsenio Spinillo

**Affiliations:** ^1^Department of Clinical, Surgical, Diagnostic and Pediatric Sciences, University of Pavia, Pavia, Italy; ^2^Department Obstetrics and Gynecology, Fondazione IRCCS Policlinico San Matteo, Pavia, Italy

**Keywords:** human papillomavirus, human immunodeficiency virus, high-grade squamous cervical lesions, squamous intraepithelial lesions, meta-analysis

## Abstract

**Background:**

This study aimed to evaluate the prevalence of multiple high-risk (HR) human papillomavirus (HPV) infections in women with human immunodeficiency virus (HIV) compared to negative controls. This study also aimed to assess the impact of multiple HR-HPVs on the risk of high-grade squamous cervical lesions (HSILs) among women with HIV.

**Methods:**

We performed a systematic search of PubMed/Medline, Scopus, Cochrane databases, and ClinicalTrials.gov from 1 January 2004 to 30 June 2023, including screenings and clinical studies evaluating the rates and role of multiple HPV infections in squamous intraepithelial lesions (SILs). Three reviewers independently screened the abstracts of the selected studies and extracted data from full-text articles. The data were subsequently tabulated and compared for consistency. The bias associated with each included study was evaluated according to the OSQE method.

**Results:**

Forty-seven studies meet definitive inclusion criteria. The quality of the observations was considered low in 26 of the included studies and moderate in 21 of the included studies. In comparative screening studies, the pooled prevalence of multiple HR-HPV was 39.1% (95% CI = 33.7–44.7) among women with (*n* = 1734) and 21.6% (95% CI = 17.3–26.1) in those without HIV infection (*n* = 912) (OR = 2.33, 95% CI = 1.83–2.97, *I*^2^ = 2.8%). The pooled ORs of HR-HPV multiple infections were similar in African (OR = 2.72, 95% CI = 1.89–3.9) and non-African countries (OR = 2.1, 95% CI = 1.46–3, *p* for difference = 0.96). Among women with HIV, the risk of HSIL diagnosed either by cytology or histology was higher among those with overall (OR = 2.62, 95% CI = 1.62–4.23) and HR multiple infections than those with single HPV infection (OR = 1.93, 95% CI = 1.51–2.46). Among women with HIV, the excess rates of multiple HPV infections and the excess risk of associated HSIL were consistent across studies including both HIV-naïve subjects and those on antiretroviral therapy, as well as in studies with different rates of immunocompromised women. When study quality (low vs. moderate) was used as a moderator, the results were unchanged.

**Conclusion:**

Multiple HR-HPV infections are common among women living with HIV and are associated with an increased prevalence of HSIL. These associations were also confirmed in studies with high rates of antiretroviral therapy and low rates of immunocompromise.

**Systematic Review Registration**: PROSPERO [registration number: CRD42023433022].

## Introduction

Both in the general population and in subjects with squamous intraepithelial lesions (SILs), multiple human papillomavirus (HPV) infections are quite common. Even though the prevalence of multiple high-risk (HR)-HPV infections in cervical cancer ranges from 4 to 19% (1), the oncogenetic mechanism underlying the role of multiple HPV infections in the development of cervical cancer in humans is still largely unknown (2, 3). Although controversial, HPV coinfection in human immunodeficiency virus (HIV)-negative women has been associated with an increased risk of high-grade squamous cervical lesions (HSILs), suggesting the possibility of synergy between multiple HR-HPVs and cervical oncogenesis (1–3). Multiple HPV infections are three times more prevalent in Africa than they are in Asia (4), indicating that racial characteristics, HIV prevalence, HPV 16 and 18 prevalence, and low vaccination rates may all be major contributors to the excess rate of multiple HPV infections in African nations (3–5). In fact, HIV infection plays a significant role in the prevalence of multiple HPV infections in Africa (5). In the general population, HIV infection is associated with an increased risk of both overall and multiple HPV cervical infections (6). Two large meta-analyses (4, 5) have shown that in Africa, multiple HPV infections are also consistently associated with an increased risk of invasive cervical cancer mainly caused by non-vaccine HPV types. On the other hand, data on the role of multiple HPVs in the risk of precancerous cervical lesions among women infected with HIV are still lacking. The primary objective of the present meta-analysis was to evaluate the pooled rates of multiple HPV infections in women with HIV compared to HIV-negative controls in both cohort and case-control studies published in the last 20 years, including all countries. The secondary objective was to assess the role of multiple HPV infections in precancerous lesions in HIV-positive patients. For this study, we considered studies of women with HIV attending screening or colposcopy services comparing the prevalence of multiple HPV infections in HSIL subjects with controls (negative or low-grade SIL as determined by cytology or histology).

## Materials and methods

### Sources

This systematic review and meta-analysis was carried out according to the suggestions of the Preferred Reporting Items for Systematic Review and Meta/Analyses (PRISMA) (7). In addition, ameta-analysis of observational studies was carried out according to standard guidelines (MOOSE) (8). The protocol, including both HIV+ and HIV-subjects, was recorded on PROSPERO on 17 June 2023 (registration number: CRD42023433022). Due to the well-known protective effect of the widespread use of highly active antiviral therapy (HAART) on the prevalence/incidence of HPV infection and SIL (9), we restricted the search to the last 20 years to avoid an overrepresentation of naïve HIV+ subjects. We searched PubMed/Medline, Scopus, and Cochrane databases from 1 January 2004 to 30 June 2023. The terms used for the searches included “Human Papillomavirus” AND “Human Immunodeficiency Virus” And “Cervical Cancer” OR “Squamous Intraepithelial Neoplasia” OR “Cervical Intraepithelial Neoplasia” OR “Cervical Dysplasia” without language restrictions. First, we included cohort or case-control studies comparing the prevalence of multiple HPV infections between HIV+ and HIV-negative subjects, irrespective of recruitment protocols and type of molecular methods used to identify HPV infection. The second objective of the search was to evaluate the prevalence of multiple HPV infections in high-grade SIL diagnosed by cytology and/or histology compared to controls (low-grade SIL or negative cytology or histology) among HIV+ subjects. To avoid over-dispersion, we included in the searches only studies with at least 5 subjects for each category studied. Three reviewers (AS, MD, and CC) screened independently abstracts of the selected studies and extracted data from full-text articles. Data were subsequently tabulated and compared for consistency. The bias associated with each included study was evaluated according to the OSQE method of Drukker et al. (10). This is a bias evaluation method developed for both case-control and cohort studies and includes several domains adapted from the Newcastle–Ottawa scale, Strobe, and ROBINS-I methods. Quality items were independently assigned by two investigators (MD and CC), and discrepancies were discussed with the other authors to reach concordance.

### Study selection

**Figure 1:**
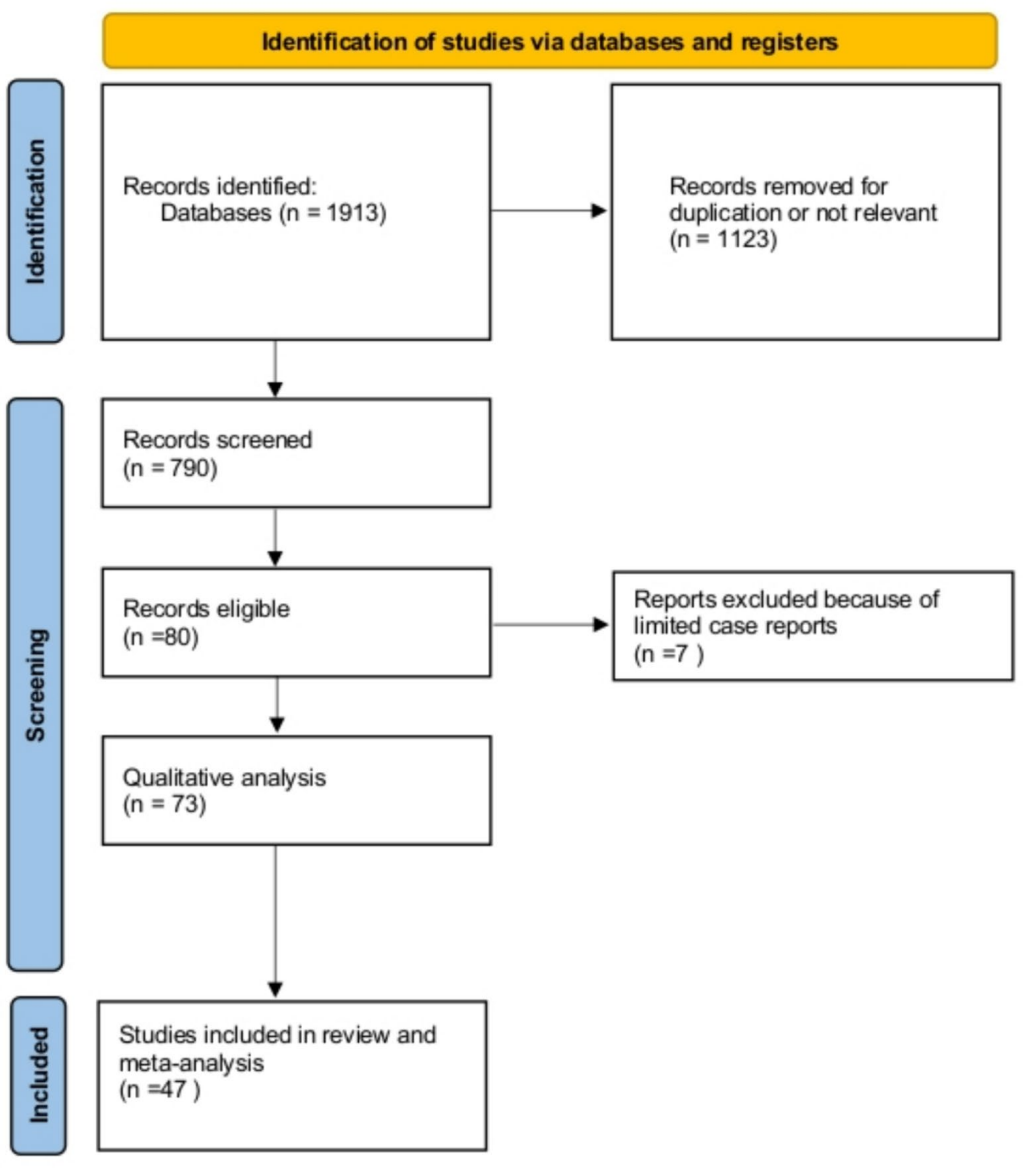
Flowchart of the study included.

A total of 1,913 studies were identified and screened for potential inclusion (Figure 1). Criteria for inclusion were as follows: (a) cohort, case-control, or cross-sectional studies evaluating the prevalence of multiple HPV infections as diagnosed using HPV-DNA molecular methods among HIV-positive and HIV-negative subjects. We included subjects enrolled in the general population or convenience samples (sex workers, women attending preventive cancer centers, or sexually transmitted disease centers); (b) cohort, case-control, and cross-sectional studies evaluating the association between multiple HPV infections and severity of cervical SILs among HIV-positive women attending colposcopic centers. We excluded studies enrolling pregnant subjects or women with invasive cervical cancer. In the end, 47 studies were declared eligible, and their data were abstracted and tabulated. The full list of the studies included is reported as Supplementary material. We used Stata (version 17; StataCorp, College Station, TX) to analyze the data. Random-effect models were used to compute pooled prevalences, odds ratios (ORs), and 95% confidence intervals (CIs) of the outcomes studied. Heterogeneity in the effects was evaluated using the *I*^2^ statistics. When heterogeneity was significant (usually at *I*^2^ > 50%), we used subgroup meta-analysis. We also tested using meta-regression for the effect of moderators such as the type of SIL diagnosis (cytology vs. histology), the type of HPV infection (HR vs. overall infection), the antiretroviral treatment (no/yes), or the rate of low (<200/mL) CD4 cell counts. Finally, we checked for publication bias (small study effects) using Egger’s test.

## Results

In 12 studies, women were tested for HIV at the enrollment (*n* = 537) (HIV naïve), whereas the remaining 35 studies included already known HIV seropositive subjects (women living with HIV). The majority of women living with HIV (median = 83%, range 50–100) were receiving some form of antiretroviral treatment at the enrollment (*n* = 3,550). HPV identification and genotyping were obtained using PCR methods in all the included studies.

**Figure 2:**
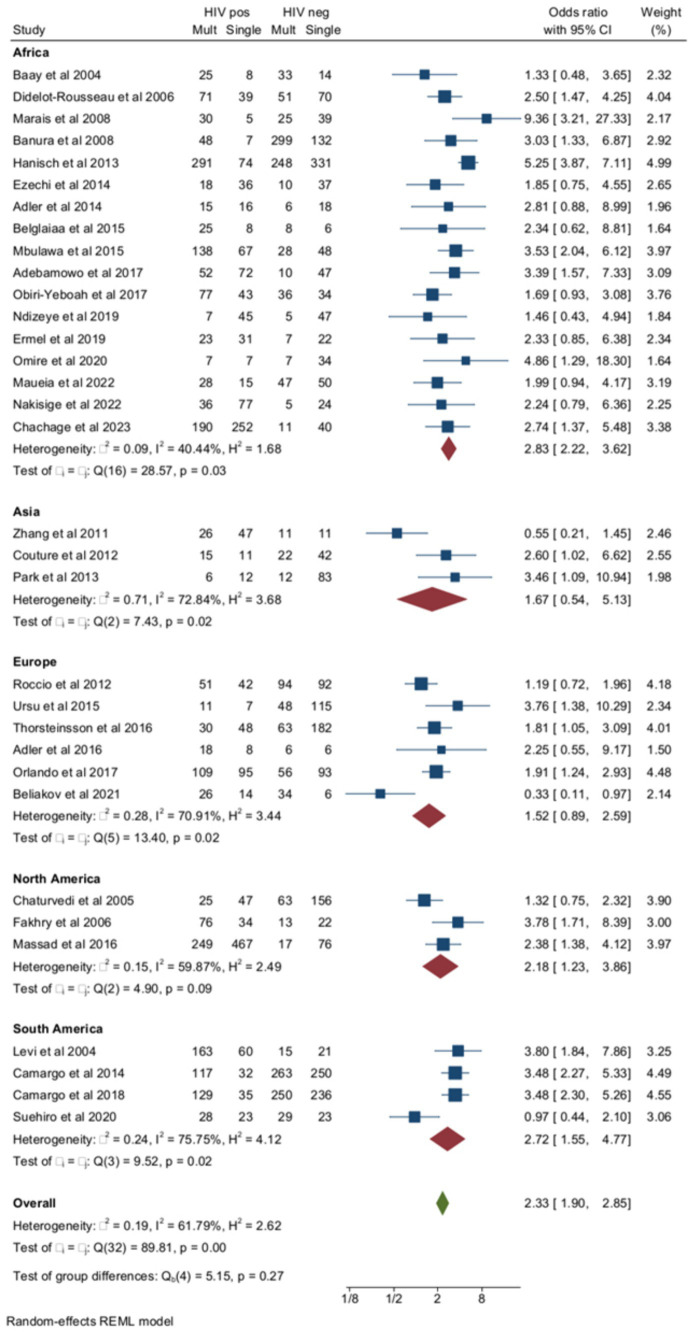
Prevalence and odds ratio (OR) of multiple human papillomavirus (HPV) infections in human immunodeficiency virus (HIV)-positive women compared to HIV-negative women.

Figure 2 reports the prevalences of multiple HPV infections in 33 of the 47 studies (18 case-control and 15 cohort studies), including 3,944 women with HIV and 4,239 negative controls. The median age of the included subjects was 35.1 years (range 12–76). In meta-regression, median age (coefficient = 0.007 ± 0.015, *p* = 0.65) did not affect the pooled prevalence of multiple HPVs. The pooled prevalence of multiple HPV infections was higher among women with HIV (56.7, 95% CI = 49.8–63.5) compared to negative controls (38, 95% CI = 32.2–43.9) (OR = 2.3, 95% CI = 1.9–2.8) and there was a rather high heterogeneity, especially in non-African countries. In subgroup analysis, pooled ORs of multiple HPV infections were 2.59 (95% CI = 1.93–3.47) in the treatment of naïve subjects (9 studies) and 2.23 (95% CI = 1.75–2.85, *p* for group difference = 0.45) in those receiving some form of antiretroviral therapy (24 studies) (Supplementary material). The relationship between multiple HPV infections and HIV positivity was studied by including the proportion of subjects with CD4 cell counts <200/mL in each study (*n* = 20) as a moderator in a meta-regression (coefficient = 1.12 ± 0.8, *p* = 0.11) and by subgroup analysis. To check if old or newer studies influenced the outcome, we included the year of the study in meta-regression. The year of the study did not influence the rates of multiple HPVs among women with HIV compared to HIV-negative subjects (beta = −0.016, *p* = 0.4). When the risk of multiple HPV infections was stratified according to the severity of immunocompromise, the ORs of multiple HPV infections were 2.36 (95% CI = 1.58–3.51) and 2.1 (95% CI = 1.66–2.58) in studies with high (≥20%) and low (<20%) rate of subjects immunocompromised, respectively (Supplementary material).

**Figure 3:**
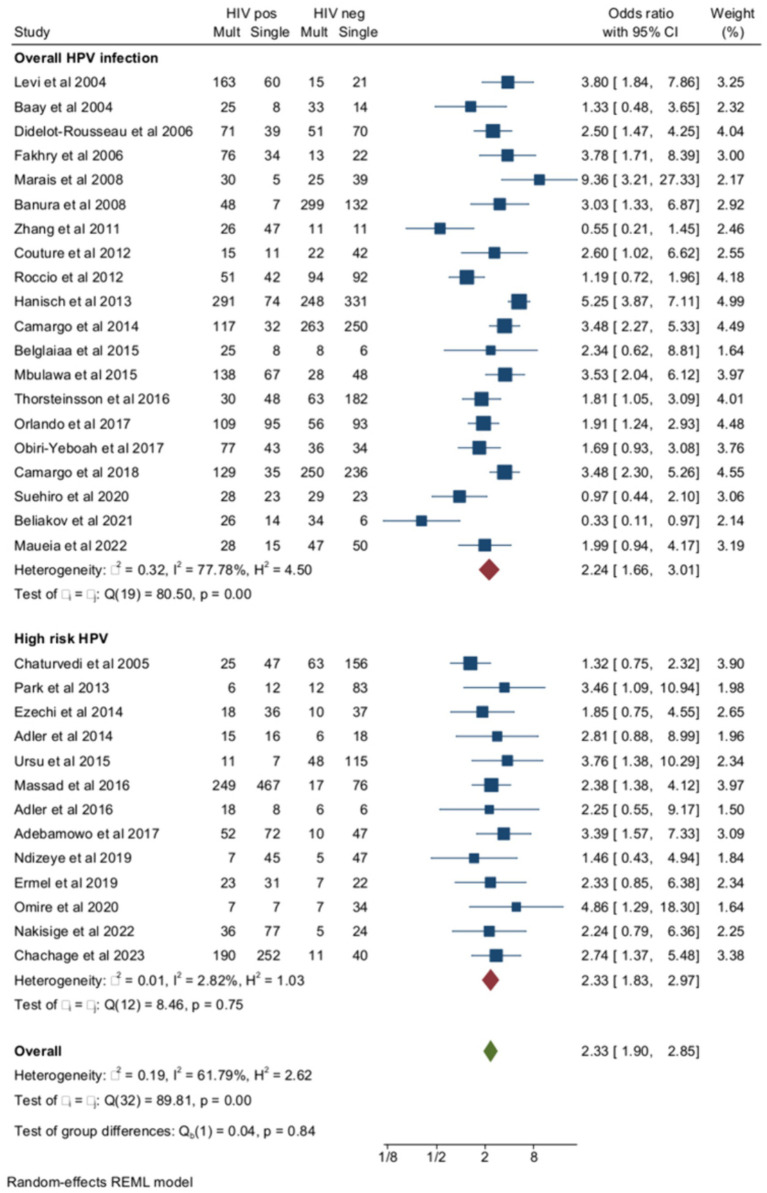
Prevalence and odds ratio (OR) of overall (high risk and low risk) multiple human papillomavirus (HPV) infections in human immunodeficiency virus (HIV)-positive women compared to HIV-negative women.

Out of the 33 studies included in the total analysis, multiple HPV infections were evaluated as overall multiple infections (both high- and low-risk HPV) in 20 studies and as HR multiple infections in the remaining 13 studies. In studies of overall HPV infection, the pooled ORs of multiple HPV infections were higher among women with HIV compared to negative controls (Figure 3). The pooled prevalence of multiple HR-HPV was 39.1% (95% CI = 33.7–44.7) in women with HIV (*n* = 1734) and 21.6% (95% CI = 17.3–26.1) HIV-negative controls (*n* = 912) (OR = 2.33, 95% CI = 1.83–2.97) (Figure 3). Although the risk of multiple HPV infections was higher among HIV+ subjects compared to HIV-subjects for both overall and HR-HPV infections, heterogeneity of the model was lower for HR (*I*^2^ = 2.8%) than overall HPV infection (*I*^2^ = 77.8%). The small study effect was not significant according to Egger’s test (Beta = −0.77, *p* = 0.33). Pooled ORs of HR-HPV multiple infection were similar in African (OR = 2.72, 95% CI = 1.89–3.9) and non-African countries (OR = 2.1, 95% CI = 1.46–3, *p* for difference = 0.96). Finally, the risk of multiple HR-HPV infections was similar among studies, including HIV-naïve subjects and those enrolling subjects receiving some form of antiretroviral treatment (Supplementary material).

**Figure 4:**
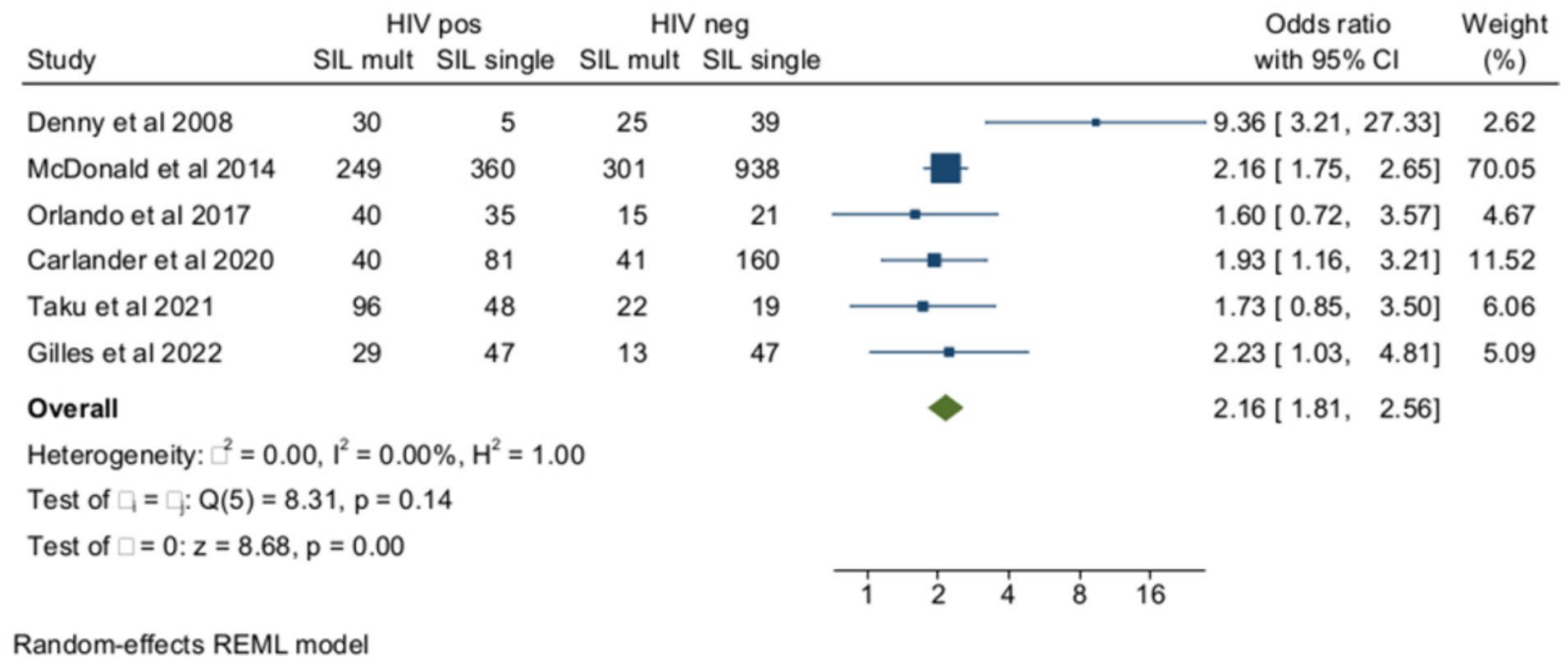
Prevalence and odds ratio (OR) of high-risk (HR) multiple human papillomavirus (HPV) infections in human immunodeficiency virus (HIV)-positive women compared to HIV-negative women.

The ORs of SIL (cytology or histology) associated with multiple HPV infections in HIV+ and HIV-subjects as opposed to unaffected controls were reported in six studies (Figure 4). The pooled prevalence of multiple HPV infections in SIL as opposed to negative controls was 52.7% (95% CI = 39.4–65.3) among women with HIV (*n* = 1,060) and 31.3% (95% CI = 23.5–39.6) (*n* = 1,641) in HIV-negative controls (OR = 2.16, 95% CI =1.81–2.56), and the heterogeneity of the model was low (*I*^2^ = 0%). Egger’s test for small studies effect was not significant (beta = −0.11, *p* = 0.9). When the analysis was restricted to histologically confirmed CIN2+ lesions (four studies), the pooled prevalence of multiple HR-HPV was 51.7% (95% CI = 40.5–62.8) among women living with HIV (*n* = 421) and 30.2% (95% CI = 23.9–36.8) in HIV-controls (*n* = 522) (OR = 2.5, 95% CI = 1.88–3.34, *I*^2^ = 0%).

**Figure 5:**
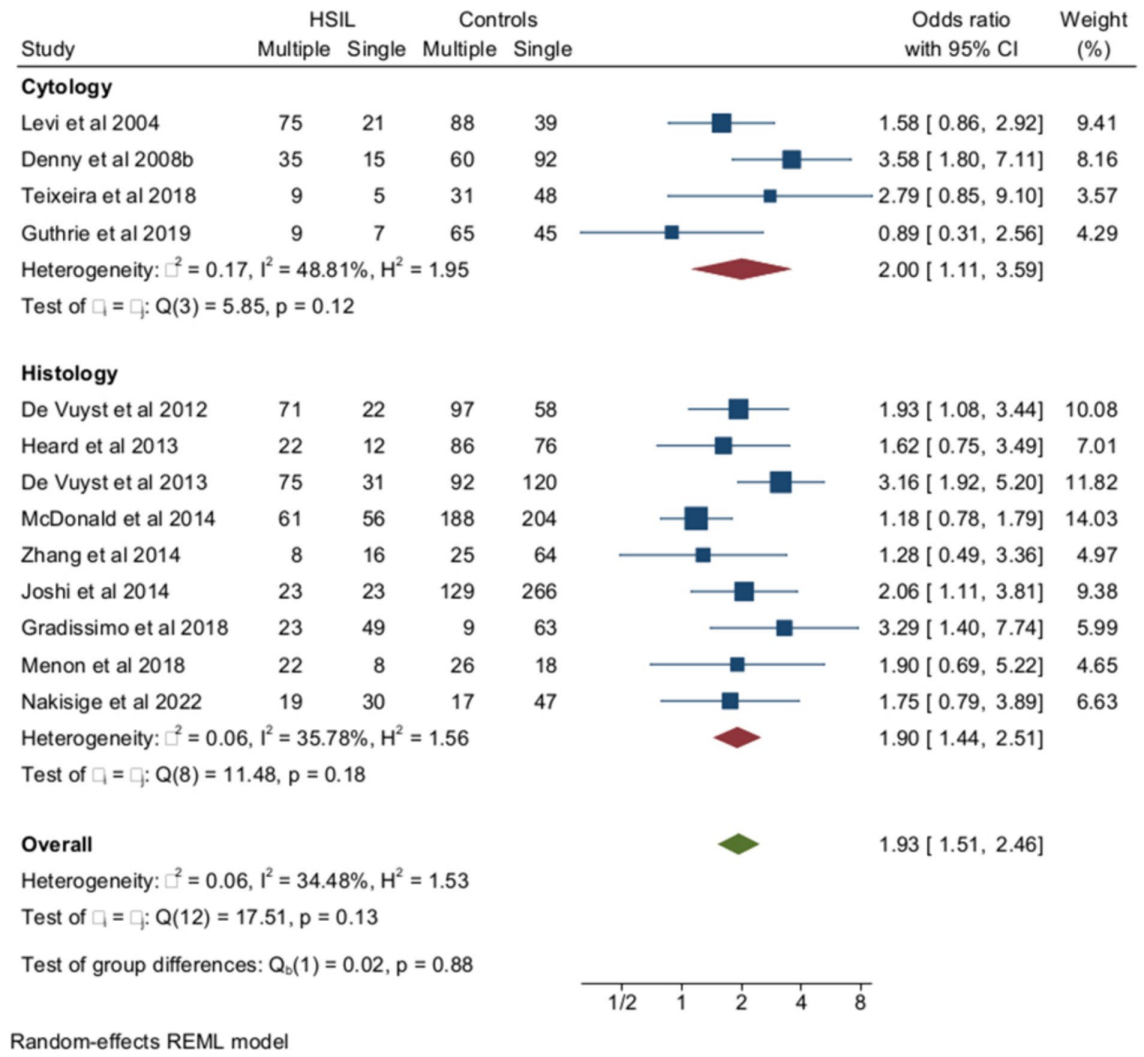
Prevalence and odds ratio (OR) of multiple human papillomavirus (HPV) infections in squamous intraepithelial (SIL) cervical lesions in human immunodeficiency virus (HIV)-positive women compared to HIV-negative women.

Among women with HIV, multiple HPV infections were more frequent among subjects with HSILs (cytology and/or histology) than in HIV+ controls with cervical cytology or histology results ≤LSIL (Figure 5). The pooled rates of multiple HPVs were 58.9% (95% CI = 49.3–68) (*n* = 747) among HIV+ women with HSILs and 43.8% (95% CI = 36–51.9) (*n* = 2053) among HIV+ controls ≤LSIL, and the heterogeneity of the model was low (*I*^2^ = 34%). When the analysis was confined to HR-HPV, the pooled prevalence of multiple HPV infections was 53.9% (95% CI = 43–64.6) (*n* = 495) and 46.1 (95% CI = 35.4–57) (*n* = 1,562) among HSILs and ≤LSIL subjects, respectively. The ORs of HSILs associated with multiple infections among women with HIV were 2.62 (95% CI = 1.62–4.23) for overall multiple HPV infections (3 studies) and 1.93 (95% CI = 1.51–2.46) for HR-HPV (10 studies) (*p*-value for group difference = 0.1). In the analysis of the effect of multiple HR-HPV on HSILs, there were five studies from Africa and five from non-African countries. The pooled ORs of HSILs were 1.43 (95% CI = 1.07–1.92) and 2.04 (95% CI = 1.42–2.94) in African and non-African studies, respectively (*p*-value for group difference = 0.14). The prevalence of HPV 16 in the 13 studies included ranged from 10.2 to 34% and, in meta-regression, HPV16 prevalence did not influence overall results (*p*-value for interaction = 0.8). The pooled ORs of HSILs associated with multiple HPV infections were 2.1 (95% CI = 1.68–2.64) in the studies (*n* = 10) including subjects receiving antiretroviral therapy and 1.7 (95% CI = 0.71–3.61) in the few studies (*n* = 3) including naïve subjects (Supplementary material). Finally, the risk of HSILs associated with multiple HPV infections was not influenced by the different rates of immunocompromised subjects enrolled in the studies examined (Supplementary material).

### Risk of bias

Tables of risk of bias compiled according to the OSQE method and including items involving representativeness, exposure, outcome, non-response, comparability, conflict of interest, and other miscellaneous potential bias factors are reported separately for cohort and case-control studies included in the analysis as Supplementary material. Given the observational nature of the included studies, the quality of the observation was considered low (higher scores in OSQE) in 26 and moderate in 21 of the included studies. Items were also included in the subsequent analysis as scores obtained by summing quality items. In the main study of multiple HPV prevalence, when the evaluation of the quality of the studies (low vs. moderate) was inserted as moderator, the ORs of multiple HR-HPV among HIV+ compared to HIV-subjects was 2.6 (95% CI = 1.74–2.74) among low-quality studies (*n* = 8) and 2.33 (95% CI = 1.83–2.97) in moderate-quality studies (*n* = 5) (*p*-value for group difference = 0.6). On the other hand, in the study of the association between multiple HPV infections and cervical disease, the ORs of HSILs were 1.6 (95% CI = 1.05–2.41) among the studies (*n* = 4) judged to be of low quality and 2.05 (95% CI = 1.53–2.76) in those judged to be of moderate quality (*n* = 9) (*p*-value for group difference = 0.4). In meta-regression, the quality of the studies included as scores did not interact significantly with the outcome measured, either as prevalence of multiple HPV infections in HIV+ compared to negative (*p* = 0.33) or prevalence of multiple infections in HSILs compared to controls (*p* = 0.21).

## Discussion

The results of this meta-analysis suggest that the prevalence of multiple HPV infections, both overall and HR types, was higher among HIV+ than HIV-negative subjects in Africa and South and North American studies. The association was not significant for studies carried out in Europe and Asia, although the heterogeneity of the analysis for these countries was high, and the number of observations was limited. The excess prevalence of multiple HPV infections was confirmed for both overall and HR HPV and was also higher among women with HIV compared to HIV-negative controls with SIL or histologically confirmed CIN2+ lesions. Finally, among women with HIV screened for cervical neoplasia, the prevalence of multiple HPV infections, both overall and high risk, was higher among those with HSILs compared to controls with cytological or histological results ≤LSIL. The excess rate of multiple HPV infections associated with HIV was similar in studies including HIV+ naïve subjects or populations with a high rate of immunocompromise and in those enrolling known HIV-positive women receiving some form of antiviral treatment. Moreover, the excess risk of multiple infections and associated HSILs was similar across studies, including women with high or low degrees of immunocompromise.

### Comparison with existing studies

Previous studies from Africa have evaluated the association between multiple HPV infections and HIV seropositivity in the general population (4, 11–13). Our meta-analysis confirms the increased risk of multiple HPV infections associated with HIV and suggests that this relationship is not limited to African studies but is also evident, although with more heterogeneity, in studies from Southern and Northern America. The association between multiple HPVs and HIV infection has been attributed to increased and prolonged exposure to HPV infection (early sexual debut and a high number of lifetime sexual partners), alongside a lower prevalence of HPV16 typical of sub-Saharan Africa (4, 14, 15). The results of this meta-analysis suggest that the association between multiple HPV cervical infections and HIV is independent of the country of origin and is similar for overall and HR-HPV cervical infections. In addition, meta-regression of published data suggests that the overall prevalence of HPV16 in the populations studied had little or no effect on the relationship between multiple HR-HPVs and overall SIL or CIN2+ lesions.

The relationship between multiple HPV infections, HIV seropositivity, and an elevated risk of cervical lesions has primarily been studied in HIV+ patients with invasive cervical cancer (5). At least two separate meta-analyses involving over 2000 cases of invasive cervical cancer from Africa suggest that the ORs of multiple HPV infections were 2–3 times greater among women with HIV than negative controls (4, 5). Interestingly, in these analyses, HPV 16 was underrepresented in HIV+ participants, while HPV 31, 35, and 68 were overrepresented in HIV-subjects (4, 5). We are not aware of any published pooled data on the connection between multiple HPV infections and cervical precancer lesions. The data from our meta-analysis indicate that multiple HR-HPV infections are linked to a higher prevalence of HSILs in women with HIV. This finding supports earlier research from Africa that multiple HPV infections could play a significant role in cervical carcinogenesis by favoring continuous and prolonged exposure to different types of HR HPVs (16).

### Quality of evidence

All the studies included in this meta-analysis were observational and had many retrospectives, so the quality of observation was low or moderate. However, the strength of the relationship between multiple HPV infections and HIV seropositivity was homogeneous across studies of low and moderate quality, suggesting that the association was consistent. Although many subjects included in the analysis were receiving some form of antiretroviral therapy, we have no data on the duration of HIV infection, which could have influenced the natural history of HPV cervical infection (16, 17). Although many studies included multiple low-risk HPVs, the increased risk of HSILs associated with HR-HPV was consistent both in African and non-African countries. Finally, the excess risk of multiple infections and associated HSILs was still evident in more recent studies, including HIV+ subjects with a low degree of immunocompromise and high rates of antiretroviral therapy.

### Biologic basis and clinical implications

Mixed low- and high-risk HPV infections are well-known markers of increased sexual exposure to infection, and, at least in African studies, the association between HIV seropositivity and multiple HPV infections has been mainly attributed to an earlier sexual debut and increased sexual promiscuity in HIV+ subjects compared to HIV-negative controls (14, 15, 18, 19). However, comparative studies between HIV-positive and HIV-negative women with similar sexual exposure (e.g., sex workers and intravenous drug addicts) (17, 18, 20, 21) found that multiple HPV infections were more common among HIV-positive than HIV-negative women, suggesting that other factors play a role in this association. In particular, several authors have described an interaction between HIV and HPV viruses on the cellular mechanism of oncogenesis, increasing the progression of HPV-associated lesions (22). On the other hand, it is also possible that HPV cervical infection could increase the susceptibility to HIV acquisition in heterosexuals by increasing the number of local target cells, such as dendritic or CD4+ cells, which are typically increased during the local immune response to HPV infection (23).

Whatever the reason for the excess prevalence of multiple HPV infections among HIV+ subjects, this association could have important clinical and epidemiological consequences.

In the papers analyzed, there was a high variation in HPV genotypes between countries. Multiple HPV infections, at least in African countries, are often associated with non-vaccine HPV types (4, 5, 24), as demonstrated by a recent meta-analysis on the prevalence of various HR-HPV genotypes in sub-Saharan African countries, which found that most of the HPVs identified are not yet included in vaccines, especially those available in that part of the world (11, 25). Similarly, high variation of HPV genotypes and high rates of multiple HPV infections have also been reported in a meta-analysis of studies from Latin America (26). Overall, all these data suggest that the variation of HPV genotypes associated with multiple HPV infections could restrict the efficacy of current vaccines, especially in countries with limited resources (26–28).

## Conclusion

The results of this meta-analysis suggest that, among women living with HIV, multiple HR-HPV infections are common and are associated with an increased prevalence of overall SIL and HSILs compared to HIV-negative controls. These associations were also confirmed in studies with a high rate of antiretroviral therapy and a low rate of immunodepression. Although the mechanism underlying the association between HIV and HPV cervical lesions is still poorly understood, it is possible that the increase in the number of genotypes associated with multiple HPV cervical lesions could negatively impact the efficacy of current vaccines, especially in low-resource nations.

## Data availability statement

The data analyzed in this study is subject to the following licenses/restrictions: the data that support the findings of this study are available from the corresponding author, (MD), upon reasonable request. Requests to access these datasets should be directed to MD, matti.domino@gmail.com.

## Author contributions

CC: Writing – review & editing. MD: Writing – review & editing. MP: Formal analysis, Writing – original draft. BG: Methodology, Writing – original draft. AS: Writing – original draft.
