# Supplementary material for: Multiple human papillomavirus infection and high-grade cervical squamous intraepithelial lesions among women with human immunodeficiency virus: a systematic review and a meta-analysis
Source: Front Med (Lausanne). 2024 Jul 15;11:1403548. doi: 10.3389/fmed.2024.1403548 (PMC11284066; doi:10.3389/fmed.2024.1403548)
Supplement: Supplementary file 1 [file Data_Sheet_1.docx]

S1: Quality of cohort studies inserted in the analysis

|  | Representativeness | Exposure | | Dependent variable | Other | Comparability | | Others | | |  |
| --- | --- | --- | --- | --- | --- | --- | --- | --- | --- | --- | --- |
| Study | Validity | valid | presence | valid | conflict | confounders | protocol | missing | modifiers | size | Quality of evidence |
| Levi et al, 2004 |  | * |  |  | * |  | * |  |  |  | low |
| Baay et al 2004 |  | * | * |  | * |  | * |  |  |  | low |
| Didelot-Rousseau et al 2006 |  | * | * | * | * |  |  |  |  |  | low |
| Denny et al 2008 (b) | * | * | * | * | * |  | * |  |  |  | moderate |
| Marais et al 2008 | * | * | * | * | * |  | * |  |  |  | moderate |
| Banura et al 2008 | * | * | * | * | * |  | * |  |  |  | moderate |
| Couture et al 2012 |  | * | * | * | * |  |  |  |  |  | low |
| Roccio et al 2012 | * | * | * | * | * |  |  |  |  |  | low |
| Heard et al 2013 | * | * | * | * | * |  | * |  |  |  | moderate |
| De Vuyst et al, 2013 | * | * |  | * |  |  | * | * |  |  | moderate |
| Park et al  2013 |  | * | * | * | * |  |  |  |  |  | low |
| McDonald et al 2013 | * | * | * | * | * |  |  |  |  |  | moderate |
| Zhang et al 2014 |  | * | * | * | * | * | * |  |  |  | moderate |
| Joshi et al 2014 | * | * | * | * | * |  |  | * |  |  | moderate |
| Ursu et al  2015 |  | * | * | * | * |  |  |  |  |  | low |
| Belglaiaa et al  2015 |  | * | * | * | * |  |  |  |  |  | low |
| Adebamowo et al 2017 | * | * | * | * | * | * | * |  |  |  | moderate |
| Gradissimo et al, 2018 |  | * | * | * | * | * | * |  |  |  | moderate |
| Menon et al 2018 | * | * | * | * | * | * |  | * |  |  | moderate |
| Teixeira et al 2018 |  | * | * | * | * | * |  |  |  |  | low |
| Guthrie et al 2019 |  | * | * | * | * |  |  |  |  |  | low |
| Carlander et al  2020 | * | * | * | * | * |  | * |  |  |  | Moderate |
| Omire et al 2020 |  | * | * | * | * |  |  |  |  |  | low |
| Taku et al 2021 |  | * | * | * | * |  |  |  |  |  | low |
| Maueia et al 2022 |  | * | * | * | * |  |  |  |  |  | low |
| Nakisige et al 2022 |  | * | * | * | * |  |  |  |  |  | low |
| Chacage et al 2023 | * | * | * | * | * |  | * | * |  |  | moderate |

Drukker M, Weltens I, van Hooijdonk CFM, Vandenberk E, Bak M. Development of a Methodological Quality Criteria List for Observational Studies: The Observational Study Quality Evaluation. Front Res Metr Anal. 2021 Jul 14;6:675071. doi: 10.3389/frma.2021.675071. PMID: 34337310; PMCID: PMC8317224.

S1: Quality of case-control studies inserted in the analysis

|  | Representativeness | Exposure | | Dependent variable | Other | Comparability | | Others | | |  |
| --- | --- | --- | --- | --- | --- | --- | --- | --- | --- | --- | --- |
| Study | Validity | valid | presence | valid | conflict | confounders | protocol | missing | modifiers | size | Quality |
| Chaturvedi et al 2005 |  | * | * | * | * |  | * |  |  |  | Low |
| Fakhry et al  2006 |  | * | * | * | * |  |  |  |  |  | Low |
| Zhang et al  2011 |  | * | * | * | * | * |  |  |  |  | Low |
| Hanisch et al  2013 | * | * | * | * | * |  |  |  |  |  | Low |
| Camargo et al 2014 | * | * | * | * | * | * |  |  |  |  | Moderate |
| Ezechi et al  2014 |  | * | * | * | * | * |  |  |  |  | Low |
| Adler et al  2014 |  | * | * | * | * |  |  |  |  |  | Low |
| Mbulawa et al 2015 |  | * | * | * | * |  |  |  |  |  | Low |
| Thorsteinsson et al 2016 | * | * | * | * | * | * |  |  |  |  | Moderate |
| Massad et al 2016 | * | * | * | * | * |  | * | * |  |  | Moderate |
| Adler et al  2016 |  | * | * | * | * |  |  |  |  |  | Low |
| Orlando et al 2017 | * | * | * | * | * | * | * |  |  |  | Moderate |
| Obiri-Yeboah et al 2017 | * | * | * | * | * | * |  |  |  |  | Moderate |
| Camargo et al 2018 | * | * | * | * | * | * |  |  |  |  | Moderate |
| Ndizeye et al 2019 | * | * | * | * | * |  |  |  |  |  | Low |
| Ermel et al  2019 | * | * | * | * | * | * |  |  |  |  | Fair |
| Suehiro et al 2020 |  | * | * | * | * |  | * |  |  |  | Low |
| Beliakov et al 2021 |  | * | * | * | * |  |  |  |  |  | Low |
| Gilles et al  2022 |  | * | * | * | * |  |  |  |  |  | Low |

Studies included in the analysis

1.Levi JE, Fernandes S, Tateno AF, Motta E, Lima LP, Eluf-Neto J, Pannuti CS. Presence of multiple human papillomavirus types in cervical samples from HIV-infected women. Gynecol Oncol. 2004 Jan;92(1):225-31. doi: 10.1016/j.ygyno.2003.10.004. PMID: 14751163

2.Baay MF, Kjetland EF, Ndhlovu PD, Deschoolmeester V, Mduluza T, Gomo E, Friis H, Midzi N, Gwanzura L, Mason PR, Vermorken JB, Gundersen SG. Human papillomavirus in a rural community in Zimbabwe: the impact of HIV co-infection on HPV genotype distribution. J Med Virol. 2004 Jul;73(3):481-5. doi: 10.1002/jmv.20115. PMID: 15170646.

3.Chaturvedi AK, Myers L, Hammons AF, Clark RA, Dunlap K, Kissinger PJ, Hagensee ME. Prevalence and clustering patterns of human papillomavirus genotypes in multiple infections. Cancer Epidemiol Biomarkers Prev. 2005 Oct;14(10):2439-45. doi: 10.1158/1055-9965.EPI-05-0465. PMID: 16214929.

4.Didelot-Rousseau MN, Nagot N, Costes-Martineau V, Vallès X, Ouedraogo A, Konate I, Weiss HA, Van de Perre P, Mayaud P, Segondy M; Yerelon Study Group. Human papillomavirus genotype distribution and cervical squamous intraepithelial lesions among high-risk women with and without HIV-1 infection in Burkina Faso. Br J Cancer. 2006 Aug 7;95(3):355-62. doi: 10.1038/sj.bjc.6603252. Epub 2006 Jul 11. PMID: 16832413; PMCID: PMC2360631.

5.Fakhry C, D'souza G, Sugar E, Weber K, Goshu E, Minkoff H, Wright R, Seaberg E, Gillison M. Relationship between prevalent oral and cervical human papillomavirus infections in human immunodeficiency virus-positive and –negative women. J Clin Microbiol. 2006 Dec;44(12):4479-85. doi: 10.1128/JCM.01321-06. Epub 2006 Oct 4. PMID: 17021055; PMCID: PMC1698387.

6.Denny L, Boa R, Williamson AL, Allan B, Hardie D, Stan R, Myer L. Human papillomavirus infection and cervical disease in human immunodeficiency virus-1-infected women. Obstet Gynecol. 2008 Jun;111(6):1380-7. doi: 10.1097/AOG.0b013e3181743327. PMID: 18515522.

7.Marais DJ, Passmore JA, Denny L, Sampson C, Allan BR, Williamson AL. Cervical and oral human papillomavirus types in HIV-1 positive and negative women with cervical disease in South Africa. J Med Virol. 2008 Jun;80(6):953-9. doi: 10.1002/jmv.21166. PMID: 18428143.

8.Banura C, Franceschi S, Doorn LJ, Arslan A, Wabwire-Mangen F, Mbidde EK, Quint W, Weiderpass E. Infection with human papillomavirus and HIV among young women in Kampala, Uganda. J Infect Dis. 2008 Feb 15;197(4):555-62. doi: 10.1086/526792. PMID: 18237268.

9.Zhang YX, Xiong Y, Gui XE, Chen SH, Rong YP, Cai HB. Epidemiologic risk profile of human papillomavirus infections in human immunodeficiency virus-positive Chinese women. Jpn J Infect Dis. 2011;64(5):411-6. PMID: 21937823.

10.De Vuyst H, Ndirangu G, Moodley M, Tenet V, Estambale B, Meijer CJ, Snijders PJ, Clifford G, Franceschi S. Prevalence of human papillomavirus in women with invasive cervical carcinoma by HIV status in Kenya and South Africa. Int J Cancer. 2012 Aug 15;131(4):949-55. doi: 10.1002/ijc.26470. Epub 2011 Nov 10. PMID: 21960453.

11.Couture MC, Page K, Stein ES, Sansothy N, Sichan K, Kaldor J, Evans JL, Maher L, Palefsky J. Cervical human papillomavirus infection among young women engaged in sex work in Phnom Penh, Cambodia: prevalence, genotypes, risk factors and association with HIV infection. BMC Infect Dis. 2012 Jul 28;12:166. doi: 10.1186/1471-2334-12-166. PMID: 22839728; PMCID: PMC3436768.

12.Roccio M, Dal Bello B, Gardella B, Carrara M, Gulminetti R, Mariani B, Spinillo A. HPV infection and intraepithelial lesions: comparison between HIV positive and negative women. Curr HIV Res. 2012 Oct;10(7):614-9. doi: 10.2174/157016212803305998. PMID: 22934657.

13.Heard I, Cubie HA, Mesher D, Sasieni P; MACH-1 Study Group. Characteristics of HPV infection over time in European women who are HIV-1 positive. BJOG. 2013 Jan;120(1):41-9. doi: 10.1111/1471-0528.12015. Epub 2012 Nov 2. PMID: 23121095.

14.De Vuyst H, Alemany L, Lacey C, Chibwesha CJ, Sahasrabuddhe V, Banura C, Denny L, Parham GP. The burden of human papillomavirus infections and related diseases in sub-saharan Africa. Vaccine. 2013 Dec 29;31 Suppl 5(0 5):F32-46. doi: 10.1016/j.vaccine.2012.07.092. PMID: 24331746; PMCID: PMC4144870.

15.Hanisch RA, Sow PS, Toure M, Dem A, Dembele B, Toure P, Winer RL, Hughes JP, Gottlieb GS, Feng Q, Kiviat NB, Hawes SE; University of Washington-Dakar HIV and Cervical Cancer Study Group. Influence of HIV-1 and/or HIV-2 infection and CD4 count on cervical HPV DNA detection in women from Senegal, West Africa. J Clin Virol. 2013 Dec;58(4):696-702. doi: 10.1016/j.jcv.2013.10.012. Epub 2013 Oct 18. PMID: 24210330; PMCID: PMC4059498.

16.Park EK, Cho H, Lee SH, Lee SG, Lee SY, Kim KH, Lee CH, Chung JS, Kwak IS. Human papillomavirus prevalence and genotype distribution among HIV-infected women in Korea. J Korean Med Sci. 2014 Jan;29(1):32-7. doi:10.3346/jkms.2014.29.1.32. Epub 2013 Dec 26. PMID: 24431902; PMCID: PMC3890473.

17.McDonald AC, Tergas AI, Kuhn L, Denny L, Wright TC Jr. Distribution of Human Papillomavirus Genotypes among HIV-Positive and HIV-Negative Women in Cape Town, South Africa. Front Oncol. 2014 Mar 14;4:48. doi: 10.3389/fonc.2014.00048. PMID: 24672770; PMCID: PMC3953716.

18.Zhang HY, Fei MD, Jiang Y, Fei QY, Qian H, Xu L, Jin YN, Jiang CQ, Li HX, Tiggelaar SM, Smith JS, Sahasrabuddhe VV, Qiao YL. The diversity of human papillomavirus infection among human immunodeficiency virus-infected women in Yunnan, China. Virol J. 2014 Dec 7;11:202. doi: 10.1186/s12985-014-0202-3. PMID: 25481842; PMCID: PMC4279793.

19.Joshi S, Babu JM, Jayalakshmi D, Kulkarni V, Divate U, Muwonge R, Gheit T, Tommasino M, Sankaranarayanan R, Pillai MR. Human papillomavirus infection among human immunodeficiency virus-infected women in Maharashtra, India. Vaccine. 2014 Feb 19;32(9):1079-85. doi: 10.1016/j.vaccine.2013.12.060. Epub 2014 Jan 4. PMID: 24397900.

20.Camargo M, Soto-De Leon SC, Munoz M, Sanchez R, Peña-Herrera D, Pineda-Peña AC, Sussmann O, Paez C, Perez-Prados A, Patarroyo ME, Patarroyo MA. Human papillomavirus detection in women with and without human immunodeficiency virus infection in Colombia. BMC Cancer. 2014 Jun 18;14:451. doi: 10.1186/1471-2407-14-451. PMID: 24942545; PMCID: PMC4067500.

21.Ezechi OC, Ostergren PO, Nwaokorie FO, Ujah IA, Odberg Pettersson K. The burden, distribution and risk factors for cervical oncogenic human papilloma virus infection in HIV positive Nigerian women. Virol J. 2014 Jan 15;11:5. doi:10.1186/1743-422X-11-5. PMID: 24433568; PMCID: PMC3896716.

22.Adler DH, Wallace M, Bennie T, Mrubata M, Abar B, Meiring TL, Williamson AL, Bekker LG. Cervical dysplasia and high-risk human papillomavirus infections among HIV-infected and HIV-uninfected adolescent females in South Africa. Infect Dis Obstet Gynecol. 2014;2014:498048. doi: 10.1155/2014/498048. Epub 2014 Oct 20. PMID: 25389377; PMCID: PMC4217359.

23.Ursu RG, Onofriescu M, Luca A, Prisecariu LJ, Sălceanu SO, Nemescu D, Iancu LS. The Need for Cervical Cancer Control in HIV-Positive and HIV-Negative Women from Romania by Primary Prevention and by Early Detection Using Clinically Validated HPV/DNA Tests. PLoS One. 2015 Jul 17;10(7):e0132271. doi: 10.1371/journal.pone.0132271. PMID: 26186361; PMCID: PMC4506070.

24.Belglaiaa E, Elannaz H, Mouaouya B, Aksim M, Mercier M, Prétet JL, Chouham S, Mougin C. Human papillomavirus genotypes among women with or without HIV infection: an epidemiological study of Moroccan women from the Souss area. Infect Agent Cancer. 2015 Dec 8;10:44. doi: 10.1186/s13027-015-0040-y. PMID:26664495; PMCID: PMC4673842.

25.Mbulawa ZZ, Coetzee D, Williamson AL. Human papillomavirus prevalence in South African women and men according to age and human immunodeficiency virus status. BMC Infect Dis. 2015 Oct 26;15:459. doi: 10.1186/s12879-015-1181-8. PMID: 26502723; PMCID: PMC4624185.

26.Thorsteinsson K, Storgaard M, Katzenstein TL, Ladelund S, Rønsholt FF, Johansen IS, Pedersen G, Hashemi L, Nielsen LN, Nilas L, Obel N, Bonde J, Lebech AM. Prevalence and distribution of cervical high-risk human papillomavirus and cytological abnormalities in women living with HIV in Denmark - the SHADE. BMC Cancer. 2016 Nov 8;16(1):866. doi: 10.1186/s12885-016-2881-1. PMID: 27821088; PMCID: PMC5100104.

27.Massad LS, Xie X, Burk RD, D'Souza G, Darragh TM, Minkoff H, Colie C, Burian P, Palefsky J, Atrio J, Strickler HD. Association of cervical precancer with human papillomavirus types other than 16 among HIV co-infected women. Am J Obstet Gynecol. 2016 Mar;214(3):354.e1-6. doi: 10.1016/j.ajog.2015.09.086. Epub 2015 Nov 14. PMID: 26433170; PMCID: PMC4775397.

28.Adler DH, Wallace M, Bennie T, Abar B, Meiring TL, Williamson AL, Bekker LG. Cumulative Impact of HIV and Multiple Concurrent Human Papillomavirus Infections on the Risk of Cervical Dysplasia. Adv Virol. 2016;2016:7310894. doi: 10.1155/2016/7310894. Epub 2016 Feb 22. PMID: 26997954; PMCID: PMC4779813.

29.Adebamowo SN, Olawande O, Famooto A, Dareng EO, Offiong R, Adebamowo CA; H3Africa ACCME Research Group. Persistent Low-Risk and High-Risk Human Papillomavirus Infections of the Uterine Cervix in HIV-Negative and HIV-Positive Women. Front Public Health. 2017 Jul 21;5:178. doi: 10.3389/fpubh.2017.00178. PMID: 28785554; PMCID: PMC5519520.

30.Orlando G, Bianchi S, Fasolo MM, Mazza F, Frati ER, Rizzardini G, Matteelli A, Zanchetta N, Amendola A, Tanzi E. Cervical Human Papillomavirus genotypes in HIV-infected women: a cross-sectional analysis of the VALHIDATE study. J Prev Med Hyg. 2017 Dec 30;58(4):E259-E265. doi: 10.15167/2421-4248/jpmh2017.58.4.804. PMID: 29707656; PMCID: PMC5912788.

31.Obiri-Yeboah D, Akakpo PK, Mutocheluh M, Adjei-Danso E, Allornuvor G, Amoako-Sakyi D, Adu-Sarkodie Y, Mayaud P. Epidemiology of cervical human papillomavirus (HPV) infection and squamous intraepithelial lesions (SIL) among a cohort of HIV-infected and uninfected Ghanaian women. BMC Cancer. 2017 Oct 16;17(1):688. doi: 10.1186/s12885-017-3682-x. PMID: 29037188; PMCID: PMC5644111.

32.Gradissimo A, Lam J, Attonito JD, Palefsky J, Massad LS, Xie X, Eltoum IE, Rahangdale L, Fischl MA, Anastos K, Minkoff H, Xue X, D'Souza G, Flowers LC, Colie C, Shrestha S, Hessol NA, Strickler HD, Burk RD. Methylation of High-Risk Human Papillomavirus Genomes Are Associated with Cervical Precancer in HIV-Positive Women. Cancer Epidemiol Biomarkers Prev. 2018 Dec;27(12):1407-1415. doi: 10.1158/1055-9965.EPI-17-1051. Epub 2018 Sep 20. PMID: 30237251; PMCID: PMC6279505.

33.Menon S, Luchters S, Rossi R, Callens S, Kishor M, Bogers J, Vanden Broeck D. Human papilloma virus correlates of high grade cervical dysplasia in HIV-infected women in Mombasa, Kenya: a cross-sectional analysis. Virol J. 2018 Mar 27;15(1):54. doi: 10.1186/s12985-018-0961-3. PMID: 29587796; PMCID: PMC5870930.

34.Teixeira MF, Sabidó M, Leturiondo AL, de Oliveira Ferreira C, Torres KL, Benzaken AS. High risk human papillomavirus prevalence and genotype distribution among women infected with HIV in Manaus, Amazonas. Virol J. 2018 Feb 17;15(1):36. doi: 10.1186/s12985-018-0942-6. PMID: 29454382; PMCID: PMC5816532.

35.Camargo M, Del Río-Ospina L, Soto-De León SC, Sánchez R, Pineda-Peña AC, Sussmann O, Patarroyo ME, Patarroyo MA. Association of HIV status with infection by multiple HPV types. Trop Med Int Health. 2018 Nov;23(11):1259-1268. doi: 10.1111/tmi.13142. Epub 2018 Sep 10. PMID: 30133078.

36.Ndizeye Z, Vanden Broeck D, Lebelo RL, Bogers J, Benoy I, Van Geertruyden JP. Prevalence and genotype-specific distribution of human papillomavirus in Burundi according to HIV status and urban or rural residence and its implications for control. PLoS One. 2019 Jun 25;14(6):e0209303. doi: 10.1371/journal.pone.0209303. PMID: 31237894; PMCID: PMC6592514.

37.Ermel A, Tonui P, Titus M, Tong Y, Wong N, Ong'echa J, Muthoka K, Kiptoo S, Moormann A, Hogan J, Mwangi A, Cu-Uvin S, Loehrer PJ, Orang'o O, Brown D. A cross-sectional analysis of factors associated with detection of oncogenic human papillomavirus in human immunodeficiency virus-infected and uninfected Kenyan women. BMC Infect Dis. 2019 Apr 27;19(1):352. doi: 10.1186/s12879-019-3982-7. PMID: 31029097; PMCID: PMC6487004.

38.Guthrie BL, Rositch AF, Cooper JA, Farquhar C, Bosire R, Choi R, Kiarie J, Smith JS. Human papillomavirus and abnormal cervical lesions among HIV-infected women in HIV-discordant couples from Kenya. Sex Transm Infect. 2020 Sep;96(6):457-463. doi: 10.1136/sextrans-2019-054052. Epub 2020 Jan 9. PMID: 31919275; PMCID: PMC884110

39.Carlander C, Lagheden C, Eklund C, Nordqvist Kleppe S, Dzabic M, Wagner P,Yilmaz A, Elfgren K, Sönnerborg A, Sparén P, Dillner J. HPV Types in Cervical Precancer by HIV Status and Birth Region: A Population-Based Register Study. Cancer Epidemiol Biomarkers Prev. 2020 Dec;29(12):2662-2668. doi:10.1158/1055-9965.EPI-20-0969. Epub 2020 Sep 23. PMID: 32967862.

40.Omire A, Budambula NLM, Kirumbi L, Langat H, Kerosi D, Ochieng W, Lwembe R. Cervical Dysplasia, Infection, and Phylogeny of Human Papillomavirus in HIV-Infected and HIV-Uninfected Women at a Reproductive Health Clinic in Nairobi, Kenya. Biomed Res Int. 2020 Jun 16;2020:4945608. doi: 10.1155/2020/4945608. PMID: 32685493; PMCID: PMC7317317.

41.Suehiro TT, Damke GMZF, Damke E, de Azevedo Ramos PLR, de Andrade Pereira Silva M, Pelloso SM, Huh WK, Franco RAF, da Silva VRS, Scarinci IC, Consolaro MEL. Cervical and oral human papillomavirus infection in women living with human immunodeficiency virus (HIV) and matched HIV-negative controls in Brazil. Infect Agent Cancer. 2020 May 11;15:31. doi: 10.1186/s13027-020-00301-y. PMID: 32426032; PMCID: PMC7216672.

42.Taku O, Businge CB, Mdaka ML, Phohlo K, Basera W, Garcia-Jardon M, Meiring TL, Gyllensten U, Williamson AL, Mbulawa ZZA. Human papillomavirus prevalence and risk factors among HIV-negative and HIV-positive women residing in rural Eastern Cape, South Africa. Int J Infect Dis. 2020 Jun;95:176-182. doi:10.1016/j.ijid.2020.02.051. Epub 2020 Feb 27. PMID: 32114194.

43.Beliakov I, Senina M, Tyulenev Y, Novoselova E, Surovtsev V, Guschin A. The Prevalence of High Carcinogenic Risk of HPV Genotypes among HIV-Positive and HIV-Negative MSM from Russia. Can J Infect Dis Med Microbiol. 2021 May 31;2021:6641888. doi: 10.1155/2021/6641888. PMID: 34122681; PMCID: PMC8189766.

44.Maueia C, Murahwa A, Manjate A, Andersson S, Sacarlal J, Kenga D, Mussá T, Williamson AL. Identification of the Human Papillomavirus Genotypes, According to the Human Immunodeficiency Virus Status in a Cohort of Women from Maputo, Mozambique. Viruses. 2021 Dec 23;14(1):24. doi: 10.3390/v14010024. PMID: 35062228; PMCID: PMC8780750

45.Nakisige C, Adams SV, Namirembe C, Okoche L, Ferrenberg J, Towlerton A, Larsen A, Orem J, Casper C, Frenkel L, Uldrick TS. Multiple High-Risk HPV Types Contribute to Cervical Dysplasia in Ugandan Women Living With HIV on Antiretroviral Therapy. J Acquir Immune Defic Syndr. 2022 Jul 1;90(3):333-342. doi: 10.1097/QAI.0000000000002941. PMID: 35195571; PMCID: PMC9203909.

46.Gilles C, Rozenberg S, Buxant F, Manigart Y, de Wind R, Houte KV, Vandenbroeck D, Delforge M, Konopnicki D. HPV genotyping in biopsies of HSIL and invasive cervical cancers in women living with HIV: A cohort- and a nested -case control study. Vaccine. 2022 Nov 28;40(50):7230-7237. doi: 10.1016/j.vaccine.2022.10.029. Epub 2022 Oct 31. PMID: 36328880.

47.Chachage M, Parikh AP, Mahenge A, Bahemana E, Mnkai J, Mbuya W, Mcharo R,Maganga L, Mwamwaja J, Gervas R, Kibuuka H, Maswai J, Singoei V, Iroezindu M, Fasina A, Esber A, Dear N, Imbach M, Crowell TA, Hern J, Song X, Hoelscher M, Polyak CS, Ake JA, Geldmacher C; AFRICOS Study Group. High-risk human papillomavirus genotype distribution among women living with and at risk for HIV in Africa. AIDS. 2023 Mar 15;37(4):625-635. doi: 10.1097/QAD.0000000000003437.Epub 2022 Dec 1. PMID: 36398743; PMCID: PMC9994804.

.


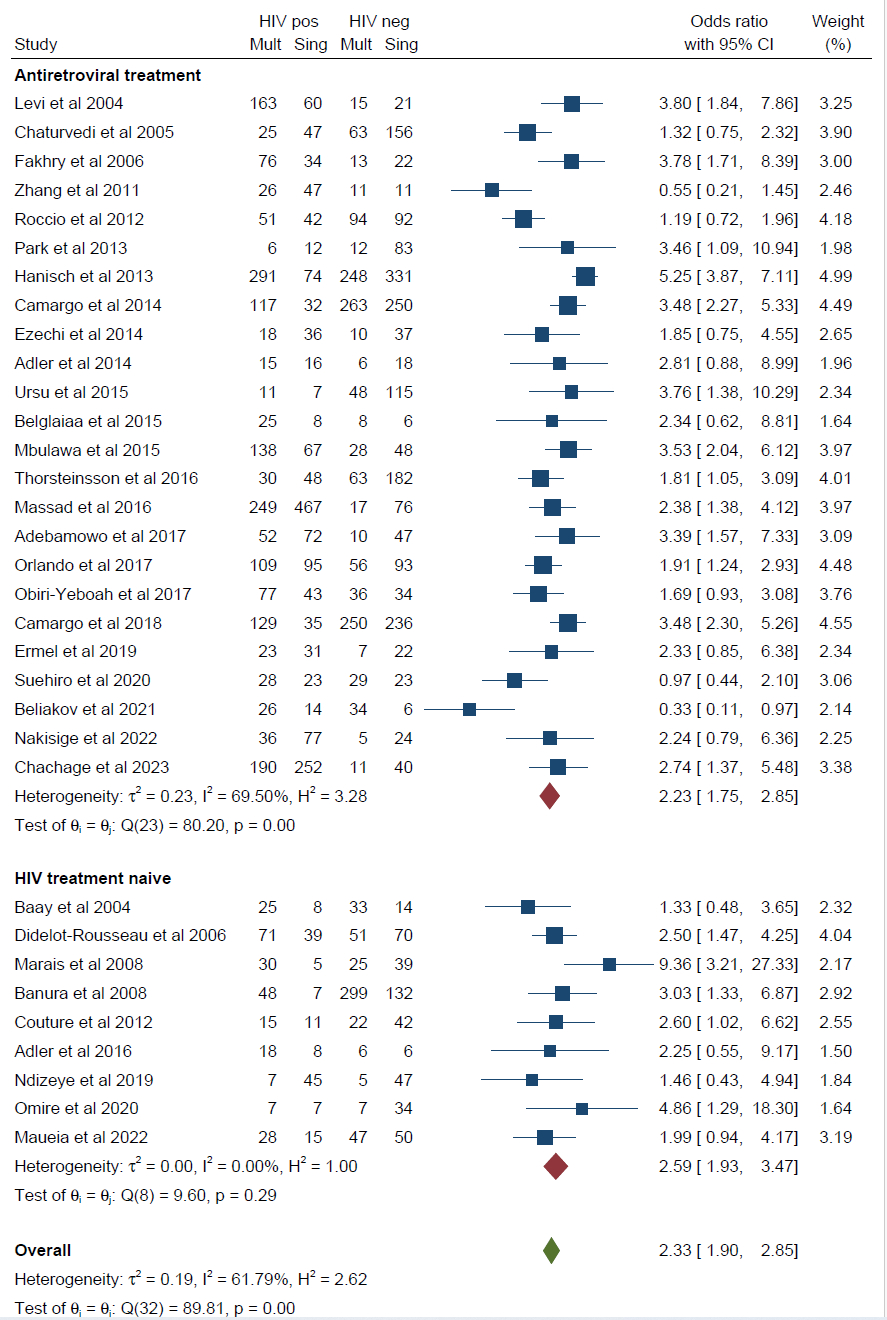


Fig 1.1 suppl: Rates and odds ratios of HPV multiple infection among HIV positive as compared to HIV negative women according to antiretroviral therapy status


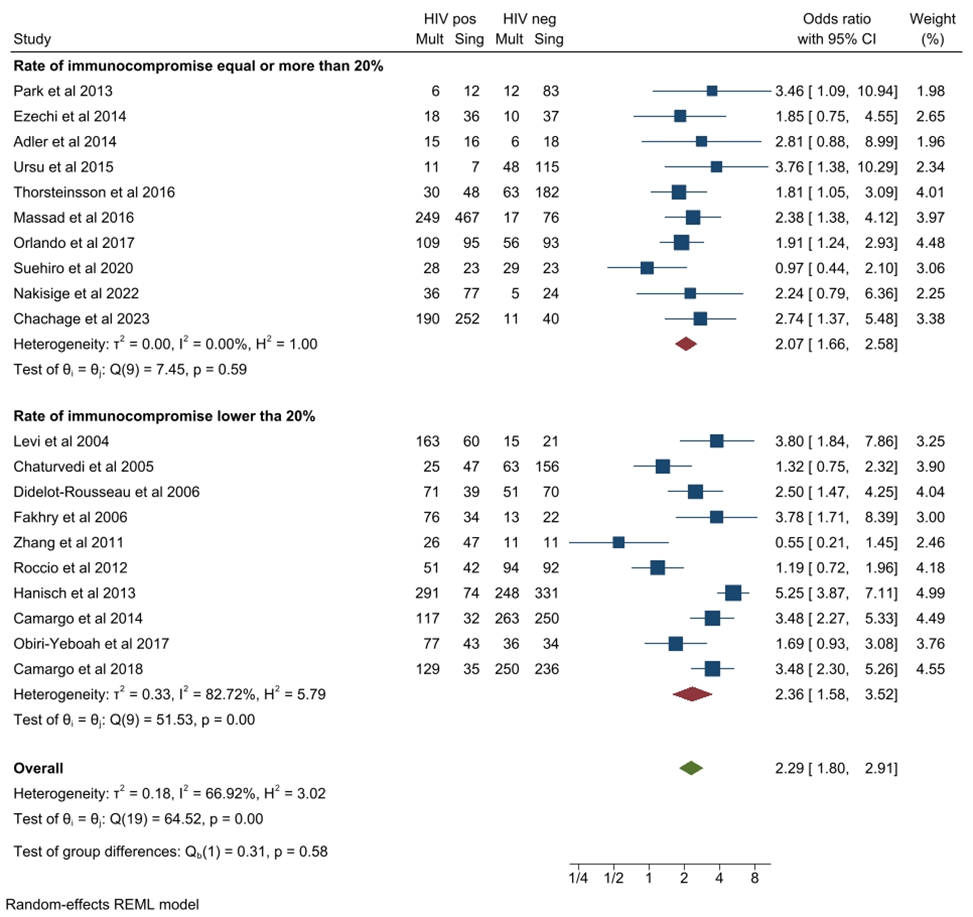


Fig.2.1. Odds ratios and 95% confidence intervals of multiple HPV infection stratified by the proportion of low (< 200 /ml) CD4 cell counts


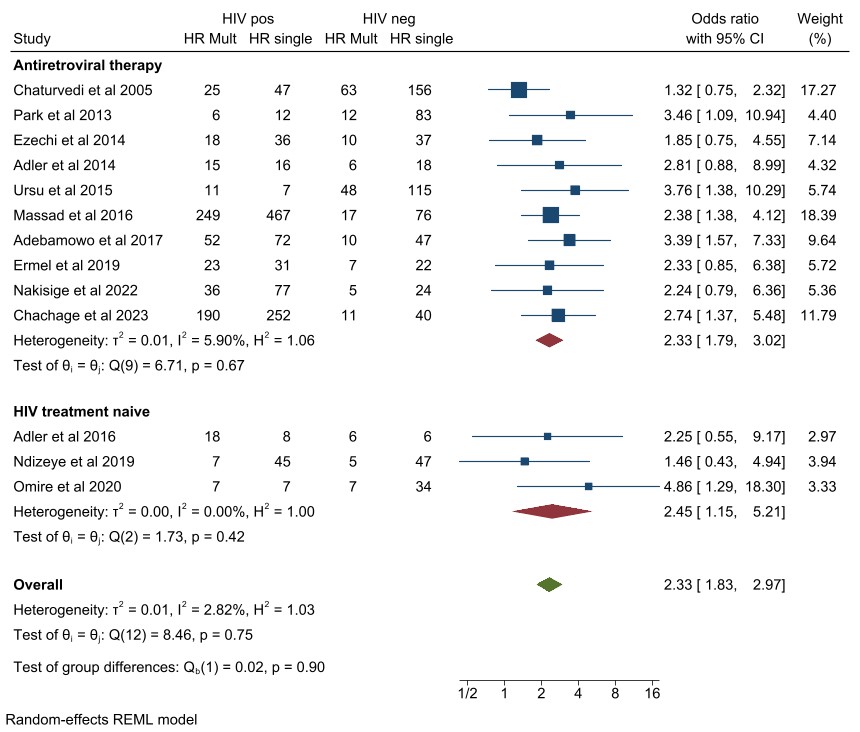


Fig.3.1: Odds ratios of multiple high risk HPV infection associated with antiretroviral therapy.


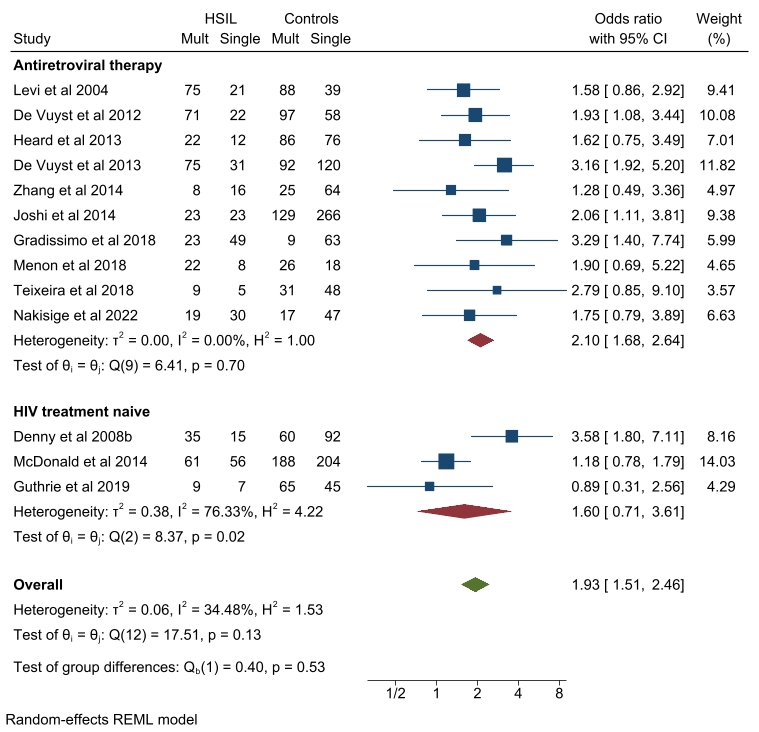


Fig 4.1: Odds ratios of HSIL associated with multiple as compared to single HPV infection among women with HIV according to antiretroviral therapy.


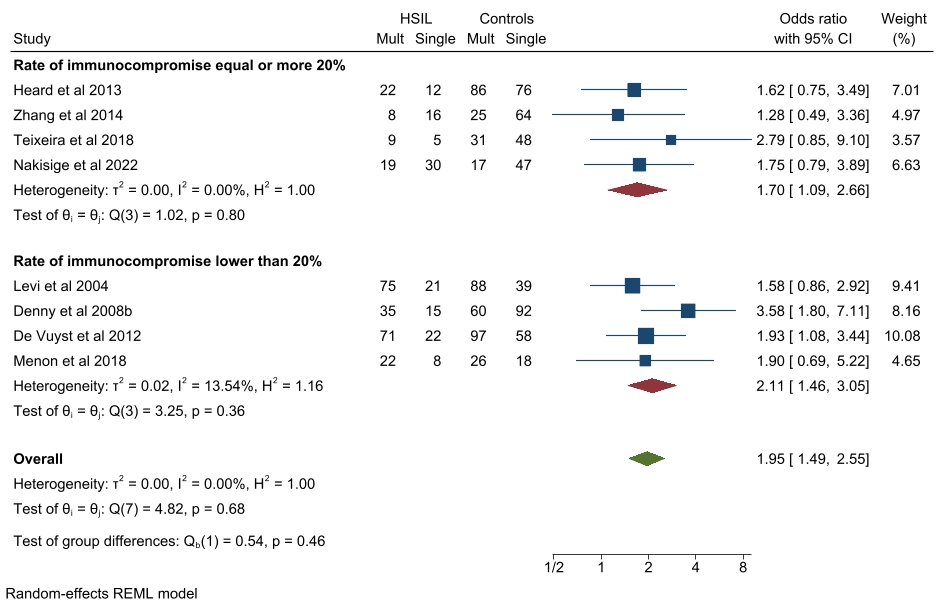


Fig 4.1: Odds ratios of HSIL associated with multiple HPV infection among women with HIV stratified by the proportion of low (< 200/ml) CD4 cell counts.
